# A “pretty normal” life: a qualitative study exploring young people’s experience of life with bronchiectasis

**DOI:** 10.1080/17482631.2021.2003520

**Published:** 2021-11-18

**Authors:** Julie Blamires, Annette Dickinson, El Shadan Tautolo, Catherine A Byrnes

**Affiliations:** a School of Clinical Sciences Auckland University of Technology, Northcote, Auckland; b School of Public Health & Interdisciplinary Studies Director-AUT Pacific Health Research Centre, Auckland University of Technology, NZ; c Department of Paediatrics; Child and Youth Health, Faculty of Health & Medical Sciences, University of Auckland, Auckland, NZ

**Keywords:** Bronchiectasis, youth, interpretive description, normal life

## Abstract

**Purpose:**

Bronchiectasis is a chronic respiratory disease that impacts significantly on quality of life for those who have it. There is a paucity of literature exploring the perspectives of children and young people. The aim of this study was to examine the day-to-day life experience of a group of young people with bronchiectasis.

**Method:**

A qualitative study using semi-structured interviews explored fifteen young people’s perspectives of life with bronchiectasis. Key themes were identified using an inductive iterative approach through constant comparative analysis guided by Thorne’s interpretive description.

**Results:**

Life with bronchiectasis was conceptualized by participants as “Pretty Normal”. This consisted of two co-existing life views which represented how young people balanced the ups and downs of adolescence while learning to accommodate the demands of living with bronchiectasis. Three key thematic elements “sore and tired”, ‘life interrupted and “looking after self”, influenced and challenged these two views of life.

**Conclusions:**

Young people with bronchiectasis portray life as being the same as their peers. Despite this, they recognized that the symptoms, interruptions, and self-management responsibilities led them to find ways of coping and integrating their experience into a new and modified view of normal.

## Introduction

Bronchiectasis is a chronic lung disease characterized by irreversible bronchial dilation, chronic inflammation, and recurrent respiratory infective exacerbations. In most developed countries rates of bronchiectasis in children and young people is low. This is partially due to a decrease in infectious diseases and improvements in childhood immunizations, nutrition, sanitation, living conditions and better access to healthcare and antibiotics (bpac nz, 2020; Lesan & Lamle, 2019). Despite this, bronchiectasis continues to be a significant health issue for children and youth, most notably in low-income countries and among indigenous communities (McCallum & Binks, 2017; Singleton et al., 2014; Telfar-Barnard & Zhang, 2019). In New Zealand (NZ), those children and young people with bronchiectasis most affected are Māori and Pacifica children and youth, and those from the lowest socioeconomic quintile (Munro et al., 2011; Twiss et al., 2005). The postulated reasons relate to social deprivation, environmental conditions such as cold and overcrowded houses, ethnicity, and recurrent respiratory infections in early childhood (Edwards et al., 2003; McCallum & Binks, 2017; Twiss et al., 2005).

Bronchiectasis in children and young people presents clinically in a similar way to adult bronchiectasis with the most common symptoms being excess sputum production, shortness of breath, chronic wet cough, and frequent chest exacerbations/infections. The focus of management is on improving both the duration and quality of a patient’s life, with the guidelines recommending regular exercise and chest physiotherapy as well as intermittent antibiotic therapy especially during times of exacerbation (Chang et al., 2021). Vigilance and a commitment to self-monitoring and maintenance of health, from both the individual and the family, is of upmost importance for children and young people as there is good evidence that disease progression can be halted with optimal clinical management(Chang et al., 2018).

Studies about children’s experience of bronchiectasis are primarily reported from the parents/caregivers’ perspectives and describe the substantial disease burden and negative impact that bronchiectasis has on psychological status and quality of life (Bahali et al., 2014; Cox et al., 2019; Gokdemir et al., 2014; Hamzah et al., 2011). Cox et al. (2019) reported a higher prevalence of both anxiety and depression among parents of children with bronchiectasis and found significantly worse self-reported, and parent-perceived health related quality of life (HRQoL), when compared to healthy peers. Key factors relating to the burden of disease such as frequent exacerbations, hospitalizations and missing out on school negatively impacted on parents/caregivers quality of life and on how they rated their child’s quality of life (Kapur et al., 2012). Although these studies give us some information about the impact of bronchiectasis on the lives of children and their parents there is a clear knowledge gap in relation to the unique experience of young people.

Qualitative studies reporting on other comparable chronic respiratory diseases provide some useful insights. Young people growing up with cystic fibrosis (CF) describe learning to cope with the symptoms, the complexity of their disease, restrictions, and self-management requirements (Kaushansky et al., 2017; Muther et al., 2018). In addition and not unlike other chronic illness among young people, depression and anxiety are linked to negative health outcomes for those with CF (Quittner et al., 2014), asthma (Vazquez-Ortiz et al., 2020) and primary ciliary dyskinesia (Behan et al., 2017). Young people with chronic respiratory disease are also at greater risk of psychosocial stress and emotional disturbance and have a poorer quality of life (QOL) when compared to their healthy peers (Behan et al., 2017; De Benedictis & Bush, 2017). Despite these challenges of living and managing a chronic respiratory disease, it has been reported that young people still strive for a sense of normalcy amidst the chaos and utilize various coping strategies to achieve this (Behan et al., 2017; Gjengedal et al., 2003).

The unique perspective of young people with bronchiectasis has not been fully investigated. Currently we do not know what matters most to young people with bronchiectasis nor have we heard their perspectives on living with and integrating bronchiectasis into daily life. Such an understanding could assist health professionals in working and planning care and could shed light on ways to improve quality of life and self-management skills of these young people. This research therefore sets out to answer the question: How do young people with bronchiectasis describe their day-to-day life and what matters most?

## Methods

In this qualitative, inductive study guided by interpretive description (ID) methodology, we explored the day-to-day life experiences of a group of young people with bronchiectasis(Thorne, 2016). Given the limited understanding of how young people experience bronchiectasis and our interest in generating knowledge relevant for the clinical or practice context, ID seemed the best fit for this study. With its roots in constructivism ID enabled us to gain insight into the subjective experiences of life with bronchiectasis (Thorne et al., 2004). Data were collected in New Zealand over a period of nine months from October 2018-June 2018.

### Participants

In keeping with ID methodology, purposive sampling was utilized to recruit a group of participants to represent different features of the population (Thorne, 2016). A spread of ages was important because of the study’s aim to focus on “young people” (as per definition outlined below); and an appropriate spread of ethnicity was important because of the significant ethnic disparities that exist in bronchiectasis rates among Māori and Pacific children and young people in NZ (Telfar-Barnard & Zhang, 2019).

Young people were invited to participate in the study if they met the following inclusion criteria:

1. confirmed diagnosis of bronchiectasis by High-resolution computed tomography (HRCT)
2. had fluency in English; and
3. were aged between 12 and 24 years of age (This age group criteria was based on the definition of the World Health Organization definition of “young people” and the age group defined as “youth” in the Government’s Youth Development Strategy (Ministry of Youth Affairs, 2002; World Health Organization, 2014).

Participants were recruited through Starship Respiratory Department and Counties Manukau Health Respiratory Service both of which are tertiary services based in Auckland and provide care for patients throughout NZ. In addition, the NZ Bronchiectasis foundation helped with recruiting participants through social media and advertising to group members. Nurse specialists, specialist physiotherapists, respiratory paediatricians and the Bronchiectasis Foundation were provided with information about the study including consent and assent forms. These intermediaries provided prospective participants with a written participant information sheet and, if the young person expressed initial interest, sought permission for the researcher to be given the participant’s contact details. Some participants were recruited using snowballing where the caregivers of participants recruited other participants for the study through their own social networks (Naderifar et al., 2017). Once the potential participant agreed to be contacted, the first author arranged for an interview date and time.

Fifteen young people of mixed ethnicity (nine females, six males) aged between 13–23 years were interviewed in this study. Basic demographic information such as gender, age, age at diagnosis and ethnicity were also collected for the purpose of describing the sample (see Table I for demographic characteristics). At the time of interview, all the young people were medically stable.

**Table I.** Participants’ demographic characteristics

Participant number	Ethnicity	Age(years)		Gender	Age diagnosis(years)
1	NZ European		13	F	8
2	NZ European		13	M	7
3	NZ European		14	F	5
4	Māori		13	M	9
5	Tuvaluan/Filipino		17	F	9
6	Māori/Cook Island		15	M	10 months
7	NZ European		16	F	6
8	Māori		13	F	4
9	Māori		13	F	4
10	Māori		14	F	9
11	Samoan		15	M	10
12	Samoan		21	F	11
13	Niuean		23	F	15
14	Tongan		15	M	10
15	NZ European		17	M	15

### Data collection

Data was collected via face-to-face semi-structured interviews. The interviews were conducted by the primary author (JB; female; Registered Nurse, lecturer). Using a topic interview guide, interviews occurred in a variety of different locations based on the participants’ wishes and included: homes of participants, hospital clinic rooms and restaurant/cafés. This was to ensure the participants were in a comfortable neutral location and was part of the trust building process. No repeat interviews were carried out and no member check occurred. Interviews lasted between 30–60 minutes and were digitally recorded. Field notes were made after each interview to capture initial impressions.

### Data analysis

In keeping with ID methodology (Thorne, 2016), data collection, coding, analysis, thematic construction, and concept development was an inductive and iterative process. Four interconnected processes enabled: examination and *comprehension* of the data elements; *synthesis* through testing and challenging initial assumptions and findings; *theorizing* and/or the creation of further themes; and finally a *recontextualization* and production of the conceptual framework (Morse, 1994; Thorne, 2016).

After each interview detailed memos on immediate understandings and personal reflections were documented. Thorne (2016) describes how making sense of the data begins the minute it is heard and continues throughout the data gathering process. In the beginning, the notes were more *descriptive*, where the participants descriptions of their experience were summarized; over time these developed and evolved into *interpretation*, where individual data was made sense of, and common threads were located. A professional transcriber transcribed all audio recordings into text. Once all the transcription files were edited to a standard word format, NVivo software® was used to store and assist with data analysis after the initial thematic analysis was completed. Immersion in the data was achieved through listening and re-listening to the recordings, reading and re-reading the data as it emerged and comparing it with initial memos and reflections.

In the next part of the analysis participant experiences were blended to describe typical patterns of behaviour or response within the data. The transcriptions were printed and read through in their entirety, to get an overall impression and identify initial themes and broad-based codes. Each transcript was read and re-read multiple times and thoughts, or clusters of ideas, were scribbled along the margins. This was not a process of line-by-line coding but rather an attempt to comprehend and highlight the significant meaning units that related to the young participants’ experience and perception of a life with bronchiectasis. Following coding each participant’s transcript, analysis moved to comparing codes and categories across the data from all participants, looking for pieces of data that had similar properties, thematic patterns or recurring ideas (Thorne, 2016). Broad, general themes were recorded first in a mind map and eventually loaded as nodes in NVivo. These initial themes and codes were reviewed multiple times by the first and second author, all the while remaining open to the possibilities in the data and constantly asking the questions, “Why is this here? What does it mean?”.

In the third part of the analysis process, initial themes and codes were developed into more refined thematic elements and codes. Mind maps and concept maps were utilized to facilitate further identification of patterns and relationships in the data. This was a lengthy process, and it took time to see the link between the thematic groupings. An important element that aided in this process was keeping in mind the practical and applied purpose of the study. This provided structure and support in decision making throughout the analysis while enabling openness to the possibilities in the data. Re-listening and re-reading while keeping the thematic patterns that had developed, helped re-affirm the interpretive fit and challenged us to keep questioning our thinking and interpretations. There were several iterations before finally reaching the final conceptual framework.

**Figure 1.**
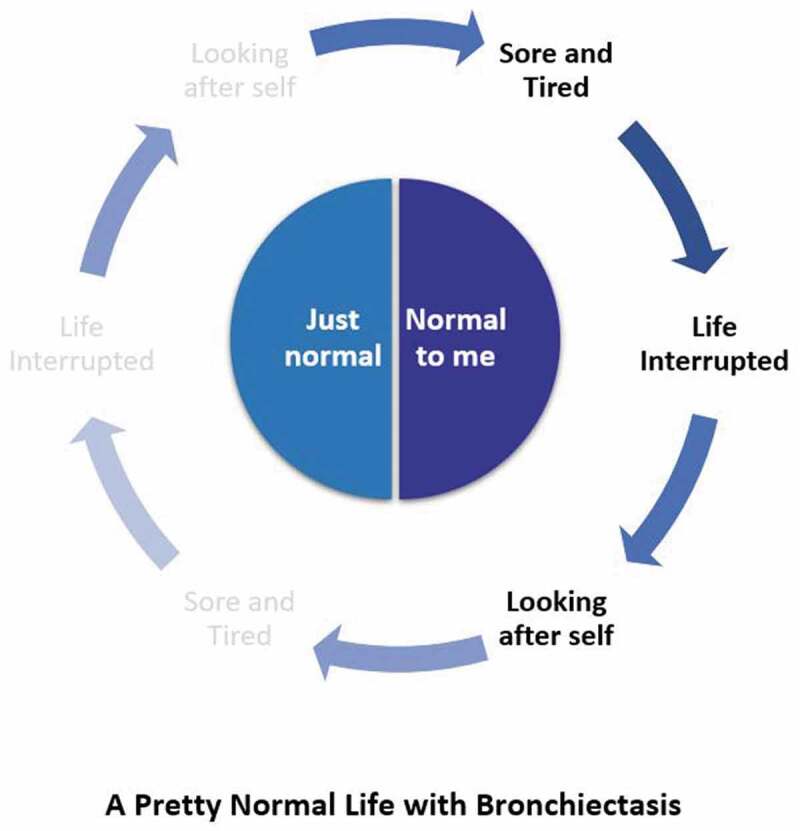
Conceptual framework of life with bronchiectasis

In the final step of the analysis the identified patterns were conceptualized into two major themes, with three encompassing thematic elements. These themes and elements were presented as a conceptual framework describing the participants life with bronchiectasis (Figure 1).

### Rigour

To ensure scientific rigour, Thorne’s principles of epistemological integrity, representative credibility, analytic logic and interpretive authority underpinned the study design, data collection and analysis process (Thorne, 2016). Epistemological integrity of this project was upheld by ensuring the design and implementation factored in the principles of ID. This involved acknowledging and recognizing experience and disciplinary preconceptions of the researchers, discussing theoretical and personal assumptions as well as critically reviewing the literature (Thorne, 2016). Representative credibility was achieved through triangulation of data sources which included the face-to-face interviews with young people with bronchiectasis, field notes and memos and extensive reflective journaling. Analytic logic is demonstrated using the four-step cognitive process framework, which outlines decision making, the audit trail and research decisions. This is outlined in detail in the data analysis section. Finally, to reflect interpretive authority, the data was interpreted considering the study’s context, as well as the first author and primary researcher checking and verifying that interview technique, data analysis and interpretation concurred with more experienced researchers within the team.

### Ethical considerations

Ethical approval was obtained from the applicable local health board, university, and research ethics committees. Written informed consent was obtained for all participants, including participant assent and parental consent for those less than 16 years old. In 10 out of 15 participants, the parent was the first point of contact; therefore, ethically, it was important to determine the young person’s competency and capacity within the context of the parental influences. In other words, to ensure that the young person was well enough informed and willing to participate rather than being pressured to participate to please their parents (Bassett et al., 2008; Moolchan & Mermelstein, 2002). In all cases there seemed a genuine willingness and desire to participate. Once the consent/assent forms were signed, they were stored in a folder in a locked office separate from the interview data. Participants selected a pseudonym. Anonymity was preserved by eliminating names and identifiers from transcripts and records.

## Findings

The findings revealed how young people initially described day-to-day life with bronchiectasis as “pretty normal”. The concept of a “*pretty normal life*” is a cognitive representation of two different views of life; “*Just Normal”* represents the view of life where they saw themselves as being just like their healthy peers, with bronchiectasis less visible and “*Normal to me”* where they accommodated and integrated a view of life that included their bronchiectasis. Three key thematic elements represent how the symptoms (Sore and Tired), interruptions (Life interrupted) and self-care responsibilities (Looking after self) encircled and influenced their view of life (Figure 1). In one view these elements are visible and strongly felt and in the other they fade into the background.

### Just normal

In the conceptual diagram “*Just normal*” holds a position as one of the two central views of life held by the participants. This relates to how they talked about the importance of being able to maintain activities that were typical of their healthy peers. In this view they did not want to be defined by their bronchiectasis. The most important thing was “fitting in” and being like their friends.

I feel like I am the same as everyone else. Just like everyone. Ah yeah, we talk, and we make people laugh. We hang out. We just do normal kid stuff. (P 14)

Participants used several strategies to achieve a personal perception of normality, and this enabled a sense of balance and satisfaction with life. For some, this involved participating in shared interests and activities with peers; for example, sports, camp, general socializing and attending church groups. Like most young people, friends were an integral and valued part of everyday life. Being with peers was about having fun and being a typical young person. In this view of life, the interruptions of bronchiectasis faded into the background.

It’s just as long as I get along with my friends and we can like do the same kind of stuff, then everything is fine and that normal (P 1)

Time spent together with friends hanging out or doing other activities enabled them to feel and see themselves as being the same as their peers. It seemed that if they were able to blend in, they were happy, and this contributed positively to their sense of wellbeing and their view of their life as “just normal”. It was important for them to not only take part in activities but also to describe their ability to be competitive among healthy peers.

I am one of the best players. I am fast as. I play just like all the boys … I am as good as any of my friends at rugby (P 6)

P 6 further emphasized that it was important to be treated the same as everyone else and they did not want to be given any special privileges. Through engaging in peer comparisons, the young participants evaluated their own situation against others in their peer group. The ability to engage in these normal or typical activities were important not only because they brought joy but also because they provided opportunity to spend time with their friends. When participants presented this view of their life, it was clear they had little interest in revealing their bronchiectasis and did not see it as central to their identity or to their day-to-day life. Participants often used words like “*it’s no big deal”* or “*you get used to it”* which signified the downplaying of their disease. The notion of life as normal was also reflected in participants future orientated goals and aspirations.

I don’t want to look back and think, I should have done that and not this and did that. Because I am doing my studies at least I will have done something with my life … (P 5)

All the participants talked about their plans and hopes such as attending university, getting a good job, “becoming famous”, having a house and/or a family of their own and ensuring that bronchiectasis did not displace these aspirations.

### Normal to me

*Normal to me* is the second central thematic element in the conceptual framework representing a modified view of life where the three key thematic elements are visible. The influence of symptoms (sore and tired), the interruptions to daily life (life interrupted) and the responsibilities of managing bronchiectasis (looking after self) forced the participants to acknowledge that the presence of bronchiectasis meant their life was not the same as their healthy peers. Through the process of accommodating the influence of the three key elements’ participants developed a new modified view of life where bronchiectasis was acknowledged, sometimes troublesome, but overall considered “*normal for them”*.

The increased visibility of the thematic element **Sore and Tired**, made it difficult to feel like a typical young person. Most participants did not even think about bronchiectasis unless they experienced symptoms, however when symptoms increased it was harder to view life as normal. Being “sore and tired” forced the young people into accommodating and paying attention to their bronchiectasis and the fact that they were experiencing a “*different kind of normal”*. Participants all developed their own individual subjective definition of “normal to me”.

I only cough in the morning see. … I am just normal I don’t really cough that much. I don’t cough every day just sometimes. (P 11)

P 11 had grown accustomed to coughing and viewed it as a background symptom. Other participants similarly also downplayed cough, with several using the expression “*I cough a bit”* or “*yeah sometimes I cough*” indicating that cough was a symptom that was present but not of great significance. For others coughing was primarily associated with getting or being sick and drew their attention towards bronchiectasis. Variations on the expressions “*only when I get a cough”* or “*unless I get sick”* were commonly used.

Symptoms presented challenges physically and emotionally for the participants and they sometimes experienced limitations in their activities. Learning to interpret what these symptoms meant for them and what actions needed to be taken was an individual learning journey that challenged their ability to live a typical adolescent life. Feelings of frustration over not being able to participate in activities, were common.

Doing PE. That’s probably the hardest. All the running that we do and keeping up with everybody … I was always at the back and couldn’t do things for very long and it was very frustrating. If I do my physio like before that it helps or take my inhaler (P 3)

This notion of an altered version of a “pretty normal life” was also seen when participants described the presence or absence of symptoms or chest infections.

I’ve only had about two to four chest infections in the last year. That’s really good for me (P 7)

The idea of two to four chest infections per year would not necessarily be considered normal or typical for most young people; however, Rose was judging this by a different scale—a scale that fitted with her view of normal as a young person with bronchiectasis. Others also talked about their bronchiectasis referring to times in the past when their bronchiectasis was more problematic, when they were younger or times when they were less stable or having and exacerbation. Their measure of how good life was, was an individual subjective experience, influenced by the visibility of the thematic element Sore and Tired.

**Life Interrupted** is the second circling theme in the conceptual diagram and represents how the participants described the intermittent disturbances that interrupted and affected many aspects of day-to-day life. It suggests a different normal state to that of their peers. Participants described physiotherapy, medications, clinic appointments and the physical symptoms of the disease as inconvenient but inevitable interruptions that temporarily stopped them from doing things they wanted to do. Participants had to learn to accommodate these interruptions into their daily routine:

I just make sure I get up early and get my breathing done. It’s a pain but that way I know I am set for the day and my chest will be clear. Stops me coughing too much at school. (P 3)

Others described how they would “*do physio before I leave for class”* or “*try to do it before showering in the morning*”. These minor adjustments in their daily routine were not insurmountable. For example, if they were a little late for an event or had to get up a bit early to do chest physiotherapy these events were considered tolerable. However, if these things got in the way of attending school, sports, family events or other social activities it was a different story.

For some people missing school would be a good thing but not for me. When people have time off school it is when they are not sick it is different when you are forced to. I tried to explain to my sister that it is not fun being off school. It is not like I enjoy it or can do anything fun and then I feel stressed about what I have missed. (P 2)

These stressful interruptions occurred for a variety of reasons including numerous clinic appointments; acute chest exacerbations requiring them to stay at home; or lengthy hospitalizations for treatments, tests and/or “tune ups” (where they received intensive antibiotic therapy). Participants talked about how the hospital was “*boring*” and how they felt isolated and disconnected from other young people. They felt discouraged and disappointed when they had to miss out on events and social activities, and this was especially felt when they were disconnected from their peers for a longer period. Coming back into the social arena of school was described as difficult and the young participants found themselves having to reintegrate back into the social group.

Being away too long was not great. I missed my friends and stuff and when I came back everyone had moved on to new groups and stuff … And I didn’t really understand it. And so that wasn’t really good in a way. (P 12)

Participants managed these interruptions by downplaying the significance. For example, when having to take medications or complete breathing exercises before going to a friend’s house, participants would describe this as “*no big deal”* or “*its just the way it is for me”*. These “abnormal” activities were integrated into daily life, and this was the way participants managed the visibility of this thematic element. Others used abstract terms to describe their bronchiectasis, such as “*my breathing problem”* or “*my lung problem”*. This downplaying of bronchiectasis provided a way of disconnecting from the seriousness of it and helped them to accommodate and view it as “*normal to them”*.

The third thematic element was named **looking after self** and illustrates how the young people were amidst a complex, transitional journey to developing self-care independence. Self-management activities such as doing physiotherapy, taking medications, attending appointments, monitoring the intermittent flares of symptoms, understanding limitations, and learning how to take ownership of their health were some of the many things that participants were learning.

This thematic element related to the young person’s evolving knowledge and belief about the self-care tasks required to look after themselves and keep themselves well. Establishing a habit and incorporating “*out of the ordinary things”* into daily life was a way in which participants redefined their notion of normal life.

I just sit there with it in my mouth while I’m playing a [video] game whatever. I got used to it, it took a while to get used to. You know wearing this weird plastic thing in your mouth. Sounding like a Darth Vadar that’s being drowned. I don’t mind it. (P 15)

Doing chest physiotherapy was seen by most as a “*nuisance*” or “*a pain”* and they described how it took up their time when they would rather be doing something else. However, despite this they admitted it they got used to it, and recognized how it helped them feel better, and potentially prevented them from getting sicker or having to go to hospital.

Well I know when I’m sick and … obviously my chest gets worse … I have my acapella and sinus rinse and all that and ways that I can clean my chest and get better. … which is really helpful now. I used to be out for a week now I can be out for a day or two. (P 14)

Experiencing positive results and recognizing how this self-care was beneficial for reducing sick time, resulted in greater adherence and a more positive attitude to self-treatment. This was influenced first by understanding the “how and why” a treatment was necessary and was apparent when participants used expressions such as “*it helps me to breathe properly”, “its supposed to clear up the lungs”* or “*it gives me healthier lungs”*. Participants of all ages expressed an understanding about why they needed to do it, even if sometimes they didn’t do it as prescribed. Participants came to know the “*right way of doing physio”* through interactions with health professionals where the most reported question was, “*Have you been doing your physio*?”. Parents were also integral to this learning experience. P 10 talked about how “*mum has been pushing me to do it regularly”* and others reported that parents would say things such as “*have you done your breathing*?” or ’*let’s do your physio now c’mon’*. The participants admitted that these reminders were what helped them to adhere to treatments.

“Looking after self” also involved learning about how to protect against getting sick. Participants described attending appointments, eating healthy food, and reducing exposure to “*bugs*” or “s*ickness”*. These protective measures were sometimes enforced by parents but over time and as they grew older, they learned to know and come to believe in them themselves.

…I try to keep myself safe so that if people are sick and coughing everywhere, I just keep away from them really. Because, if they cough on me I’ll be sick the next day, I know for sure. It’ll be okay for others but it will be 10 times worse for me because I have bronchiectasis and all of these symptoms will come out (P 13)

Other important aspects that underpinned the thematic element, “looking after self”, involved making choices, taking control, and learning to share in decision making. This was a complex process where participants were encouraged by parents to develop autonomy through role-modelling and practice.

Mum asks me if I want to … if I want to go into the hospital. Or if I want to try and manage it at home. And I always say manage it at home ….so yeah like lots of physio, exercise, sleep and eating well … Yeah. Its better home … not missing out on anything at home or with friends yeah. But if I don’t get better then I have to go [to hospital] (P 6)

P 6’s parents were helping him to gain independence with decision making within a safe and supportive environment, where he could share in the decision making that concerned his bronchiectasis management. Developing confidence in making choices and taking control was part of the way in which participants moved towards self-care independence. The participants in this study were all at different stages of this learning progression. When parents and health professions supported independence, provided opportunities to share decision making and planned transition to adult services this positively influenced the shift to autonomous self-management for the young participants.

### Discussion

The findings of this study support the conceptual framework of life for young people with bronchiectasis as a “pretty normal life”. In the conceptual diagram (Figure 1) “just normal” holds a central place and describes how feeling normal and fitting in with peers was the most important thing for the young participants. This quest for normalcy is supported by other studies related to young people with chronic illness, not wanting to be defined by their illness (Ferguson & Walker, 2014; Horky et al., 2017; K. MacDonald, 2017; Shearer et al., 2013; Waldboth et al., 2016). Participants used several strategies to achieve normality, and their perception of “just normal” was individual and personal. For some it was reflected in their ability to participate in shared interests and activities with peers; others used social comparison describing how capable or *more* capable they were at certain activities. These are common strategies used by young people, with and without chronic illness as part of normal adolescent development (Heaton, 2015; Ragelienė, 2016). It is typical that during adolescence young people will self-compare with peers as they try to figure out who they are and where they fit. Participants were keen to talk about their friendships, school achievements, family and their future aspirations suggesting they were journeying through adolescence similarly to their healthy peers. They had little interest in revealing their bronchiectasis and did not see it as central to their identity or to their day-to-day life. Studies in young people with CF have also found that young people do not view themselves as sick but instead view their life as ordinary, despite the assumptions of those around them that their disease would be more central to their self-image (Horky et al., 2017; M. Macdonald et al., 2019). This may relate to the lifelong nature of CF, these young people have grown up knowing no other life, so it is indeed viewed as ‘just normal”. This appears relevant to young people with bronchiectasis who similarly may have never known a life without the disease.

Despite the participants initial assertion that their life was “*just normal*”, they admitted to a second view of life influenced by the presence of symptoms, self-management responsibilities and the interruptions bronchiectasis imposed on their lives. This in keeping with other research, highlighted how young people learn to normalize (and get used to) “out of the ordinary” aspects of their disease, and contribute to how young people cope with chronic illness (Bailey et al., 2018; Kirk & Hinton, 2019; M. Macdonald et al., 2019; Matthie et al., 2016). The current study acknowledges an individualism to the normalization process, with participants developing individual subjective definitions of what “normal” and “doing well” looked like for them. They therefore normalized life with bronchiectasis in different ways.

Participants described four key areas of life that were frequently interrupted by their disease: school, social interactions, sport, and family. Many described how missing school was the most stressful thing about having bronchiectasis. Lengthy and multiple appointments, as well as hospitalization for acute exacerbations were the most cited reasons for school absence. School is a big part of normal life. Missing it was not only socially disruptive, but it also had a negative impact on academic goals and achievements. These findings fit with previous studies related to young people with chronic illness (Geist et al., 2003; Kaffenberger, 2006; Shaw & McCabe, 2008; Sturge et al., 1997). Disruptions to school, became increasingly significant as participants progressed through higher school years. Participants related this to increased pressure and stress associated with secondary school. Previous studies have also found this to be the case with more missed appointments occurring in older adolescents and young adults, with interference with school being the most common reason (Chariatte et al., 2007; Neal et al., 2001). While we found frustration over missing events that occurred at school, there was no evidence that participants missed appointments because of school commitments. This may relate to the fact that most participants were of an age where parental influence on attendance at clinic was still strong.

The young people minimized the social impact of bronchiectasis and this contributed to their ability to cope and feel less overwhelmed by their illness. Knafl and Deatrick (1986) described minimizing the adjustment that having a chronic illness requires, as a cognitive strategy used by young people. The young people used normalization as a strategy to accept the reality of living with bronchiectasis while not allowing it to dominate their life. The notion of “fitting in”—being normal—and the adaptive process of normalization while living with chronic illness is particularly salient for young people because of the influence of peers, the social pressures related to this developmental stage, and the young person’s desire to maintain a typical life.

Other authors have also described how families adapt behaviours to create normal family life within the context of having a child with a chronic condition or severe physical disability (Emiliani et al., 2011; Gantt, 2002; Knafl et al., 2010; Morse et al., 2000; Rehm & Bradley, 2005). These studies acknowledge parents play an integral role in teaching their children how to cope, adapt and learn to normalize their condition, as we have found here.

The young participants in this study were on their own individual journey towards self-care independence and learning to look after themselves as a young person with bronchiectasis. They demonstrated resiliency as well as the capability to adapt and integrate bronchiectasis self-management into their pretty normal life. This study identified two key findings related to transitioning to self-management. Firstly, all young people demonstrated capability to self-manage and secondly this was an individual journey influenced by parental willingness to support and hand over care.

All the participants described in various ways how they monitored and were aware of their symptoms and understood the need for the necessity of treatments. Richard and Shea (2011) described how self-management includes both self-monitoring and symptom management and, importantly, the management of functional, emotional, and psychosocial consequences of having a chronic health condition. Most participants in our study had knowledge about their illness and recognized changes in their condition and when to seek help. Knowledge about their bronchiectasis, as well as knowledge about risks and symptoms, translated to positive action in relation to self-management. The age variability of the young people in the participant group supports the view of other researchers that young people have this capability across a range of ages not only in later adolescence (Lindsay, Kingsnorth, & Hamdani, 2011). As Sawyer & Aroni (2005)) eloquently pointed out “young people do not magically develop the capacity for self-management on their 18th or 21st birthday” (p.406)().

The transition to self-management is an individual journey supported by parental involvement and willingness to share management. All the participants no matter where they were on this trajectory were still reliant on family members for support. Strong family relationships and high levels of family functioning have been shown to encourage young people to engage in self-management (Modi et al., 2012). Similar to other studies our findings did not find any common personal characteristics that either hindered or supported this transition among participants; instead, it was good balance of individual actions and parental support and encouragement that contributed to self-management development (Karlsson et al., 2008; Young et al., 2019).

### Limitations

This study may not be generalizable to other young people with bronchiectasis in other countries. There is also the potential that the experience may have been different for young people newly diagnosed or who had acquired the condition in late childhood. Future research comparing young people newly diagnosed versus those who have had the diagnosis since birth would provide further insight and identify if there were notable differences. At the time of the interviews, all the participants (except for one) were well and this could have influenced the participants’ responses in the interviews. If done during a period of ill health their experiences and perceptions as presented may have been different.

### Practice implications

This study has shown that young people do not view bronchiectasis as the most important thing in their world. It is important that healthcare professionals working with these young people are aware of their wider concerns and interests beyond the diagnosis and treatment of bronchiectasis. To engage successfully with young people consideration should be given to the individual perception of the illness as well as their aspirations to live a “pretty normal” life.

Young people as consumers of healthcare have specific needs. This study highlighted how many of the interruptions to day-to-day life were a result of healthcare-imposed obligations such as attending clinic appointments or prolonged hospitalizations. This is a significant source of frustration and disruption to the lives of young people. Consideration should be given to how healthcare services are delivered to minimize impact on a school day, so they meet the needs of young people and their families. The World Health Organization’s (1999) five criteria (accessibility, acceptability, appropriateness, effectiveness, and equity) for assessing the adequacy and quality of health services for adolescents could provide a useful starting framework for evaluation of current and future services.

This study highlighted the unique and individual and very personal nature of the condition. This reminds healthcare professionals of the need to be mindful of these differences and to work collaboratively with the young person and their family to individualize care. This is of importance in supporting the development of self-management skills prior to transitioning to adult services. Communicating with and uncovering the needs of each young person provides a challenge for health professionals often due to workload, time restraints and funding limitations. However, early planning and a transition process that occurs over time in collaboration with the young person and their family, means a successful and individualized transition process should be possible (Dickinson & Blamires, 2013).

This study provides insights into the capability of young people to self-manage and has important implications for practice. Assessment of self-management skills of young people with bronchiectasis is crucial for supporting the successful transition to adult care and adulthood. In addition, these findings have highlighted the role of parents in the transition towards shared management. Health professionals have a critical role to play in supporting parents and young people to learn how to share and develop the young person’s skills in care management. Strategies such as education with families about how and when to commence this shift of responsibility as well as advocating shared decision-making as soon as developmentally appropriate, appear to be well supported by the young people in this study.

### Conclusion

This research has shown how young people with bronchiectasis experience day-to-day life as *pretty normal* adapting with the demands of the disease. A conceptual framework shows how these young people concurrently balance two separate views; one in which they were normal and living the same life as their peers, “*just normal*”, and a second view, “*normal to me*”, which encapsulates how they accommodated their bronchiectasis. It shows the importance of thinking in a more holistic way which incorporates the young person’s subjective personal experiences. It demonstrates the importance of their participation in care planning, treatment and management as well as designing services which minimize the interruptions to their lives. The findings also provide an important reminder to health professionals that first and foremost young people value being normal. They have the same concerns and stresses about school, friends, and family as any other young person and these are the things that matter most. Having a chronic illness such as bronchiectasis undoubtedly challenges these important matters, but it is only a small part of how young people view their life.
